# 3-Dimensional Physiologic Postural Range of the Mandible: A Computerized-Assisted Technique—A Case Study

**DOI:** 10.1155/2013/698397

**Published:** 2013-10-01

**Authors:** Todd Shewman

**Affiliations:** Noraxon USA, 3630 Ashridge Street, Columbus, OH 43219, USA

## Abstract

Previous studies demonstrated that while the mandible assumes its resting position in space, antagonistic muscles should assume minimal muscle activity within a spatial range. This zone of mandibular rest has been mapped using physiologic parameters of muscle activity and incisal spatial kinematics. This case study expands on previous research by monitoring incisal and posterior jaw position and includes lateral pterygoid muscle activity, thus allowing for determining the spatial range including additional relevant coordinates and muscle activity. Four positions were evaluated: a maximum physiologic open position, a maximum physiologic closed position, physiologic rest position, and maximum physiologic protrusion position. Within the physiologic zone of rest formed by these 4 positions, the vertical and anterior borders of the envelope of function may be documented for the incisal and posterior mandible in true 3-dimensional fashion to assist the clinician in determining a physiologic interocclusal freeway space and vertical dimension of occlusion. Advantages and limitations are discussed.

## 1. Introduction

Clinical application of a maxillomandibular orthopedic relationship including nerves, muscles, ligaments, dental occlusion, and both temporomandibular joints for optimal function can be challenging. However, failure to consider these basic dynamic elements of dental occlusion in providing temporomandibular dysfunction (TMD) treatment may result in a functional compromise. Functional compromise may either predispose or ultimately lead to muscle pathology, destruction of the dentition, supporting structures and/or the temporomandibular joints. Even mild forms of compromise result in dysfunction of the neuromuscular apparatus and associated structures [1].

In an age that demands evidence-based care, combining objective measurements of physiologic and anatomic components is a natural evolution from treatment based on subjective and mechanical concepts, to computer-assisted technologies when seeking objective answers to complex physiologic functions [2].

## 2. Mandibular Posture

Posture may be defined as the relative position of the various parts of the body with respect to the egocentric coordinate system, the exocentric coordinate system, and the geocentric coordinate system [3]. Postural control involves the meaningful integration of many different neural systems that control postural components, including those associated with cognition. The mechanical problem of maintaining the posture of any single region of the body is that there are profound effects on proximal and distal regions. 

The orientation of any body part, such as the mandible, may be described in terms of these frameworks and it reflects a dynamic process.

The habitual postural position of the mandible when at rest in the upright position and the condyles are in a neutral unstrained position in the mandibular fossae is also called “postural position” [4]. Anatomical rest position, or loose packed position, for the TMJ is with the mouth slightly open so the teeth are not in contact [5]. 

Through the use of surface electromyography (SEMG), muscle activity of the masticatory system can be objectively measured. The repeatable postural range at minimum baseline SEMG levels has been shown to be consistent with physiologic rest position and jaw posture [6]. Physiologic rest of the mandible, therefore, may be defined as the minimum amount of muscle activity required to maintain anatomical rest. 

To achieve physiologic rest, the masticatory and cervical muscles must be balanced and coordinated with respect to left and right sides and the muscles of the posterior neck must be in balance with the anterior cervical muscles. This represents a stable mandibular position concurrent with a stable head position [7, 8] as only a few millimeters of increased inter-occlusal distance may influence a large reduction of masticatory muscle activity [9]. Therefore, interocclusal distance and muscle activity are intimately related and physiologically relevant. The postural range of the mandible should be a repeatable zone where the mandible may return allowing the investing musculature to function from physiologic rest [7, 8].

Additional research supporting SEMG clinical techniques in dentistry are numerous and conveniently provide guidelines where SEMG may be employed within treatment approaches including appliance therapy [10–19], orthodontic intervention [20–24], and dentures [25, 26].

## 3. Surface Electromyography (SEMG) and Jaw Kinematics in Determining a Mandibular Postural Position

**Figure 1:**
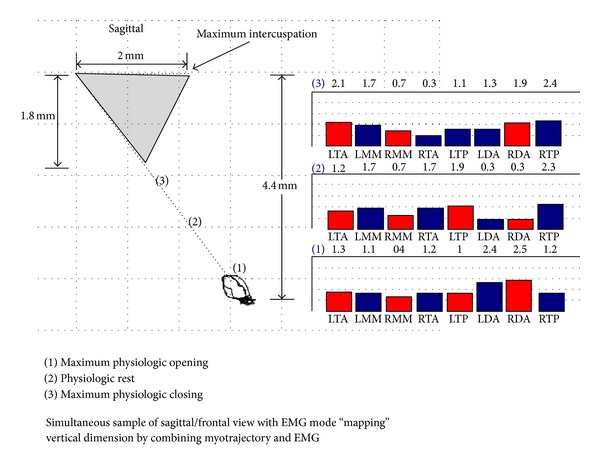
Adapted and modified from [26, page 9]. SEMG of the temporalis anterior (TA), masseter (MM), temporalis posterior (TP), and suprahyoid (DA) sites in combination with incisal spatial kinematics. “Maximum physiologic opening” or the bottom of the physiologic zone (incisal position 1) was determined when the suprahyoid activity was above what was determined as normal levels. The top of the zone or “maximal physiologic closing” (incisal position 3) was determined when elevator activity increased to greater than what was determined as physiologic normal levels. Physiologic rest (incisal position 2) position after 45–60 minutes of ULF-TENS.

Through the use of incisal based, magnetic kinematic technology and SEMG, Mazzocco et al. [27] proposed a clinical protocol by which an incisal vertical physiologic envelope may be mapped in the TMD patient (Figure 1).

A postural envelope defines the limits of stability in space in all directions. As long as the sway envelope stays within the limits of stability, balance is maintained. If sway exceeds these limits, strategies involving neighboring regions must be employed to regain balance. If an individual's center of gravity is more anterior, posterior, or lateral than center, a smaller sway envelope is tolerated before losing balance [28]. The demand of neighboring systems then increases to remain within this smaller physiologic envelope. Therefore, if mandibular rest position or mandibular postural sway exceeds the zone of the physiologic resting range, a combined strategy including those of neighboring regions (e.g., the cervical spine, etc.), must be incorporated in an effort to maintain stability. Unfortunately, once the system's adaptive capacities have been surpassed, pathology would ultimately develop.

A stable, physiologic occlusion would consider the spatial position from which the postural resting position of the mandible is maintained within a spatial zone that offers relatively minimal, balanced muscle activity in order to be able to provide symmetrical and prompt activation of the jaw musculature as the teeth are brought into occlusion, where maximal muscle recruitment is available and where the mandible may return to the physiologic resting range promptly and symmetrically.

Previous clinical works have been limited to incisal vertical dimension and lack documentation of posterior jaw position and an anterior boundary. Knowledge of the anterior border would enable clinicians to establish a jaw position within a boundary which, if exceeded, would require the neighboring systems to accommodate to maintain stability. Furthermore, posterior mandibular position is also of great importance as loss of posterior occlusal support leads to noticeable cranial condyle movement [29] as well as significantly altered effects on cervical muscle activity and spinal curvature with asymmetrical posterior occlusal support [30]. Therefore, it would enable the clinician to objectively ascertain if the chosen mandibular position from the anterior and posterior coordinates is within vertical and anterior physiologic boundaries.

## 4. Lateral Pterygoid 

The concept that disturbance to the activity of the lateral pterygoid muscles plays an important role in the etiology of TMD and is widely accepted; however, there is a dearth of scientific evidence to support this clinical notion and the role of the lateral pterygoid muscle in normal function remains controversial. 

Motor unit studies at computer tomography confirmed that sites demonstrate and confirm that the inferior head of the lateral pterygoid (IHLP) and the superior head of the lateral pterygoid (SHLP) are active during opening, protrusion, and contralateral jaw movements, while a minority of SHLP were active during closing movements. These percutaneous results also provide clinically valuable information and have demonstrated that the SHLP and IHLP were minimally active when the jaw was in the clinically determined postural jaw position [31–33].

The presence of elevated activity in the lateral pterygoid muscle at resting jaw posture may be of clinical significance in that variations in the level of lateral pterygoid muscle activity could influence and/or assist in determining the anterior positioning of the condyle in relation to the disc and eminence, as well as the positioning of the jaw at postural position.

Through the works of Hiyama et al. [34, 35] and Thomas, [36] it has been shown that through intraoral recording techniques, it may be possible to monitor the activity of the lateral pterygoids using surface electrodes. Although the nature of the surface electrode disallows distinction between the activities of the superior and inferior heads of the lateral pterygoid muscles, this provides tremendous promise for clinical purposes. 

In this case study, the physiologic window of postural rest position inclusive of anterior and posterior mandibular position relative to the maxilla and muscle activity of the intraoral lateral pterygoid placement was explored.

## 5. Materials and Methods

Case study design including a 43 year old male with normal occlusion, with temporomandibular dysfunction. The subject was a unilaterally involved combined myogenous and arthrogenous patient (groups I and II according to RDC/TMD) [37] and reported moderate to severe muscular pain at rest and during mandibular movements on the right side; pain was also associated with palpation of the TMJ area. Pain was classified as grade I-II according to the chronic pain classification scale (low disability with low to high intensity pain). Clicking (in opening/closing) or articular crepitus, on the right side were subjectively found.

Additional objective data included right joint sounds analysis that revealed possible crepitus, adhesions, scarring, and degenerative issues. Imaging results revealed slight osteogenic degeneration, sclerosis, and posterior and superior displacement of the right condyle. 

SEMG data was collected from the bilateral temporalis anterior (TA), masseter (MAS), and suprahyoid (DIG) sites using pregelled bipolar Ag/AgCl surface electrodes (Noraxon USA) with a diameter of 1 cm and an interelectrode distance of 2 cm center to center, parallel to muscle fiber direction over the muscle belly as recommended [38]. The reference electrode was placed on the C-7 spinous process. All SEMG electrode sites were rigorously abraded and cleansed with sterile alcohol prep pads prior to electrode placement.

The activity of the bilateral lateral pterygoid (LP) sites was recorded intraorally by way of surface electrodes per Hiyama et al. [34, 35]. The electrodes used were a small Ag/AgCl surface electrode Blue Ambu N with a monitoring area of 15/28 mm^2^ and a decentralized snap. The electrodes were trimmed and adhered to reach an interelectrode distance of approximately 1 cm and secured on the mucosa in the buccal vestibule distal to the maxillary tuberosity with an adhesive. Prior to electrode placement, the mucosal tissue was dried using a standard paper towel and then cleaned with a sterile alcohol prep pad.

All SEMG data was collected with a telemetry device, preamplified and band-pass filtered in hardware between 20 and 500 Hz (Noraxon USA) with input impedance greater than 100 Mohms and sampled at 1000 Hz with an overall gain of 1000. Data was rectified and smoothed at 100 ms (movag) in WinNMBite software (Zebris GmbH). 

**Figure 2:**
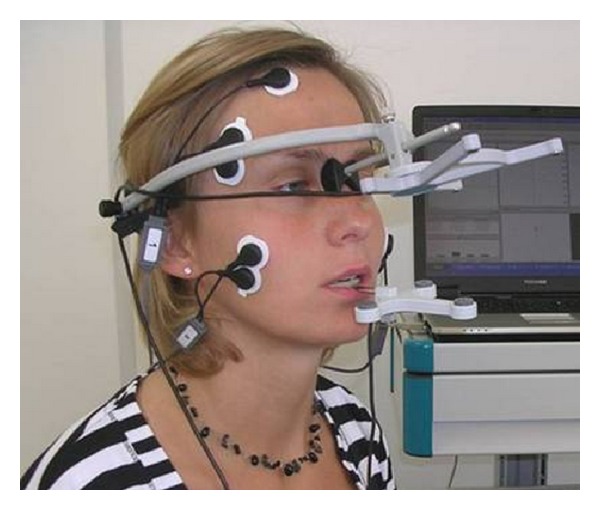
Jaw Motion Analysis system mounted on subject. Courtesy of Zebris Medical GmbH.

Spatial analysis of the lower jaw was determined in conjunction with SEMG using the Jaw Motion Analysis system (JMA) from Zebris Medical GmbH (Figure 2). The JMA system has been determined to be accurate for recording and evaluating jaw motion [39–41]. 

The JMA utilizes ultrasonic sensors attached to a light head frame (approximately 3.9 ounces) and emitters (weight—approximately 10 grams) that were attached to the mandibular dentition via Stomahesive (Convatec Inc). The resolution of the ultrasonic path is approximately 0.085 mm with a maximum sampling rate of 200 Hz (sampling rate was 75 Hz for the case study).

Documentation of the subject's maxillary right and left distobuccal molars and the incisal points were determined using the pointer system. The lower jaw is then referenced to these three points in space. These coordinates used are relative to the maxillary plane and provide incisal and posterior mandibular coordinates relative to the maxilla.

**Figure 3:**
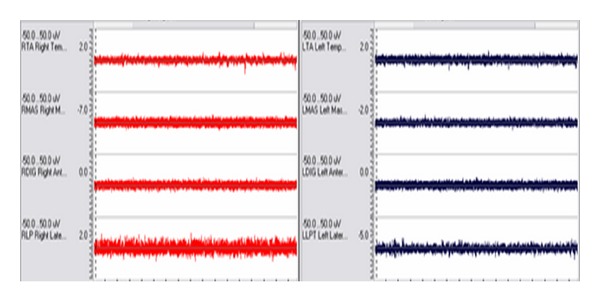
SEMG activity of the bilateral temporalis anterior (TA), masseter (MAS) suprahyoid (DIG), and lateral pterygoids (LP) at the initial baseline/postural resting position (Pre-TENS).

The subject was seated upright comfortably in a straight-back chair without head support, with the Frankfurt occlusal plane parallel to the floor. After SEMG electrode placement was completed, baseline/postural resting values of each site were recorded (Figure 3).

**Figure 4:**
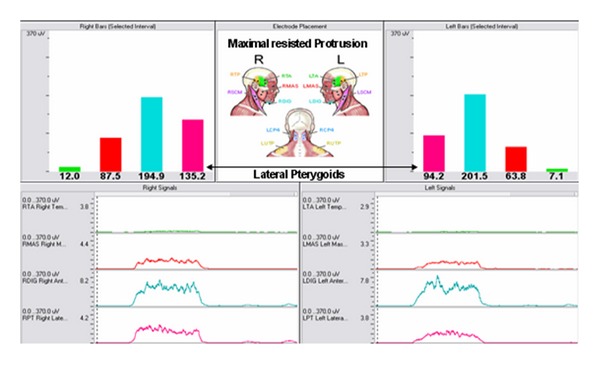
Recorded during jaw protrusion against resistance while the teeth are separated. Baseline values of the lateral pterygoids were normalized to this maximal effort.

Following the baseline recording, the subject was instructed on how to perform a maximal voluntary contraction (MVC) for the masseter, temporalis anterior, and pterygoids. SEMG baseline/postural data of the temporalis anterior and masseter sites were normalized to their corresponding mean SEMG values recorded during a two second maximal clench. With the teeth apart where maxillary and mandibular incisal position was even, a recording during a 2 second maximally resisted protrusive effort was performed (Figure 4). SEMG baseline/postural data of the lateral pterygoid sites were normalized to their corresponding mean SEMG values recorded during the two second maximum resisted protrusion effort.

**Figure 5:**
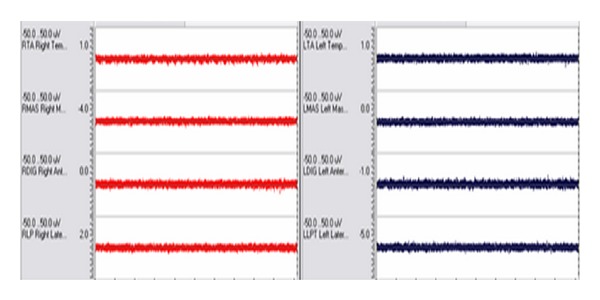
SEMG activity of the bilateral temporalis anterior (TA), masseter (MAS) suprahyoid (DIG), and lateral pterygoids (RLP/LLPT) after 25 minutes of ULF-TENS. Recording demonstrates that baseline levels from all sites are low and balanced.

Ultralow frequency transcutaneous electrical stimulation (ULF-TENS,) was chosen as the relaxation method due to research demonstrating the efficiency in relaxing masticatory muscles [42–44]. Following application of ULF-TENS, (Dolotens, Neuromuscular Technologies, Inc.) for 25 minutes, the subject was seated in a comfortable head-supported upright position with the Frankfort horizontal plane parallel to the floor and an additional baseline recording was taken to ensure that relaxation of all muscles was achieved (Figure 5). Then, the physiologic rest position was determined utilizing a traditional protocol as described by Hickman et al. [45].

**Figure 6:**
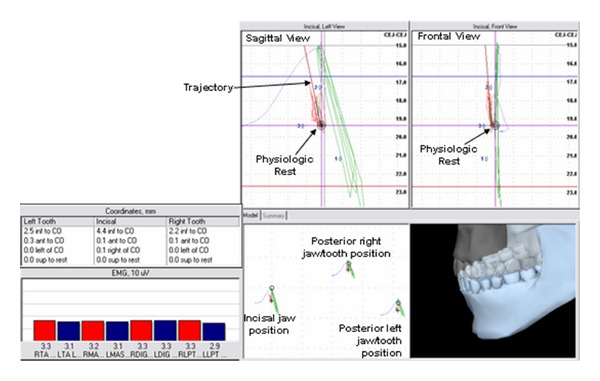
Spatial position of the lower jaw combined with bilateral SEMG activity of the temporalis anterior (TA), masseter (MAS), suprahyoid (DIG), and lateral pterygoid (LPT) sites. Coordinates listed are representative of mandibular position relative to centric occlusion and physiologic rest.

Physiologic rest position of the mandible may be described as a position assumed by the mandible within the physiologic window (Figure 6). This position was identified after appropriate SEMG values were established to maintain balanced, relatively minimal muscle activity of the jaw elevator and depressor muscles.

**Figure 7:**
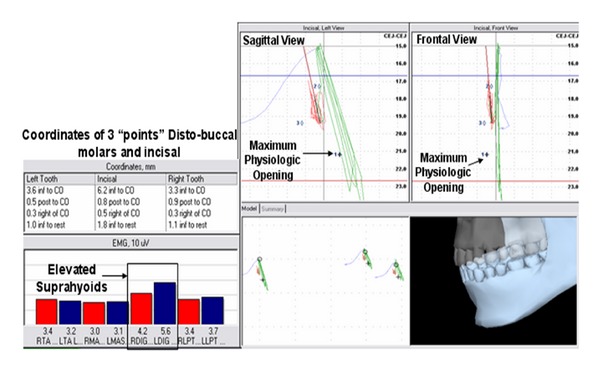
Spatial position of the lower jaw combined with bilateral activity of the temporalis anterior (TA), masseter (MAS), suprahyoid (DIG), and lateral pterygoid (LPT) sites. Coordinates listed are representative of mandibular position relative to centric occlusion and physiologic rest and were documented during the “burst” of suprahyoid (DIG) activity, and determined to be “Maximum Physiologic Opening”.

As the masticatory muscles are stimulated to contract, under the influence of ULF-TENS, the mandible is propelled through interocclusal rest (freeway space) forming an extended computed trajectory (Figure 6). The first spatial position/border, “maximum physiologic opening” was identified based upon SEMG parameters using similar methods described by Lynn and Mazzocco (Figure 7) [19]. Maximum physiologic opening was obtained by having the subject slowly open the mouth until suprahyoid or lateral pterygoid SEMG activity demonstrated a consistent elevation in excess of physiologic rest activity. The subject was instructed to perform this motion 3 times to evaluate consistency. The spatial position when either the lateral pterygoid or suprahyoids demonstrated a consistent “burst” of activity above physiologic rest values was documented (Figure 7).

**Figure 8:**
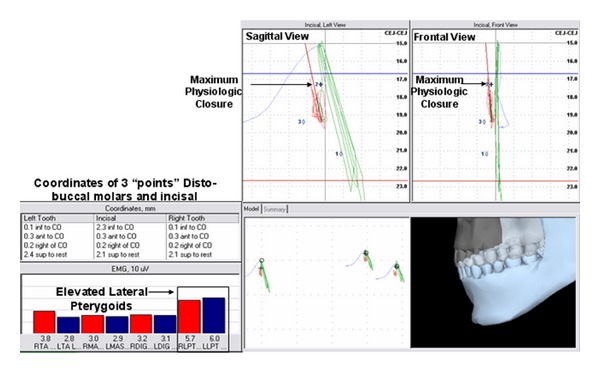
Spatial position of the lower jaw combined with bilateral activity of the temporalis anterior (TA), masseter (MAS), suprahyoid (DIG), and lateral pterygoid (LPT) sites. Coordinates listed are representative of mandibular position relative to centric occlusion and physiologic rest and were documented during the “burst” of LPT activity and determined to be “Maximum Physiologic Closing”.

The second position, “maximum physiologic closure” was identified based upon resting SEMG parameters and has been described by Mazzocco et al. [27]. Maximum physiologic closure was obtained by having the subject to slowly close the mouth until it was determined that the elevator muscle activity (masseter, temporalis anterior, or lateral pterygoids) demonstrated a consistent elevation in excess of physiologic resting activity and greater than 2% of their MVC. The threshold of 2% was chosen based on previous work that demonstrated a mathematical calculation of normal subjects having a resting baseline of less than 2% of MVC for the mandibular elevators [19]. Acceptable baseline amplitudes, may be operationally defined as less than a 1% to 2% MVIC amplitude for tasks that are functionally performed for about 1 hour or longer (e.g., posture of the mandible) [46]. The subject performed this motion 3 times to evaluate consistency. The spatial position where the lateral pterygoid, masseter or, temporalis anterior sites demonstrated a consistent “burst” above 2% MVC was documented (Figure 8).

**Figure 9:**
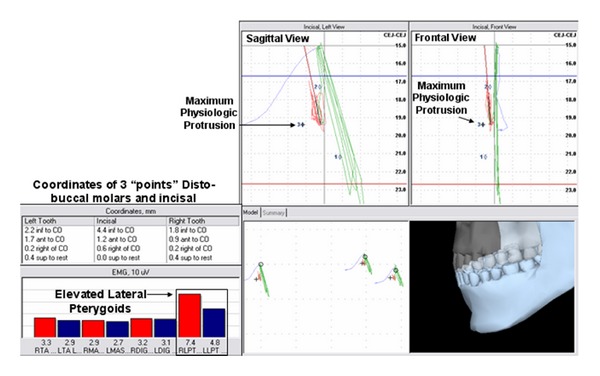
Spatial position of the lower jaw combined with bilateral activity of the temporalis anterior (TA), masseter (MAS), suprahyoid (DIG), and lateral pterygoid (LPT) sites. Coordinates listed are representative of mandibular position relative to centric occlusion and physiologic rest and were documented during the “burst” of LP activity and determined to be “Maximum Physiologic Protrusion”.

A third position, “maximum physiologic protrusion” (Figure 9) was identified based upon resting EMG parameters and was obtained by having the subject to slowly push the jaw forward (protrude) from physiologic rest position until the mandibular elevators or lateral pterygoid activity demonstrated a consistent elevation in excess of physiologic rest activity (greater than 2% MVC).

## 6. Results

Spatial position for incisal, right and left posterior jaw positions relative to their corresponding physiologic positions are presented in Table 1.

Physiologic rest was spatially determined when all elevator muscle levels were at relatively minimal levels and balanced. Incisal vertical demonstrated the greatest discrepancy. When establishing a spatial position of “maximal physiologic opening”, the suprahyoids demonstrated an initial elevation in activity in 2 of the 3 efforts. The 2nd effort demonstrated a simultaneous response of the right lateral pterygoid and suprahyoids. When establishing a spatial position of “maximal physiologic closing”, the lateral pterygoids consistently demonstrated a bilateral elevation in activity prior to the temporalis anterior and masseter recording sites in all 3 trials. When establishing a spatial position of maximal physiologic protrusion, the right lateral pterygoid site was consistently the first to demonstrate an elevation in activity over all muscles, in all 3 trials.

## 7. Discussion

As previously indicated, once adaptive capacities have been surpassed, pathology develops. This is obvious within the masticatory and associated structures. Shortened muscles can initiate pain by affecting intramuscular nociceptors [47] or through metabolic effects [48]. As muscles shorten, tension increases and concurrent mechanical stress and pain may be induced by additional tension on tendons, joints, ligaments, as well as occlusion of the vascular bed prohibiting metabolic turnover. Tenderness within these tissues contributes to the sensitization of the associated neurons lowering the pain threshold. This nociceptive input arises from muscular hypertonicity and fatigue. Therefore, treatment directed towards muscle components that limit muscle hypertonicity can decrease pain related to the dysfunction present [26]. 

**Figure 10:**
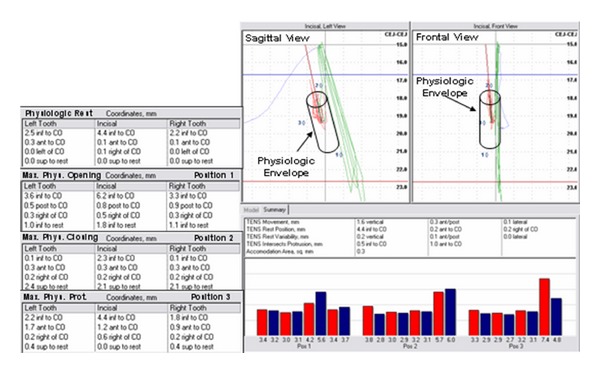
Spatial position of the lower jaw combined with bilateral activity of the temporalis anterior (TA), masseter (MAS), suprahyoid (DIG), and lateral pterygoid (LPT) sites. Coordinates listed are representative of mandibular position relative to centric occlusion and physiologic rest, maximal physiologic opening (position 1), maximal physiologic closing (position 2), and maximal physiologic protrusion (position 3).

In the case study presented, each spatial position was documented when there was evidence of muscle activity above clinically acceptable baseline levels (hypertonicity). Therefore, the resting physiologic envelope of postural jaw function in this subject would be slightly superior to maximal physiologic opening, slightly inferior to maximal physiologic closing, and posterior to maximal physiologic protrusion, and it approximates the computed trajectory to be within a physiologic muscular position (Figure 10).

The data would suggest that in this subject, an increase in vertical dimension may be considered to be compatible within the physiologic range. The vertical dimension compared to anterior/posterior positioning may require the largest change to reach a homeostatic state with mild differences between right and left posterior mandibular positions. If vertical dimension changes were to be considered on this subject, the vertical dimension would allow relatively moderate changes (less than 3.6 mm left and 3.3 mm right posterior jaw positions, resp.) and suggest mild changes in 3-dimensions to the subject's right in 3 of the 4 positions documented. Also, any anterior change may be limited in this subject as all positions were less than a millimeter anterior to CO to be within the physiologic range. These data suggest that the vertical dimension of both the anterior and posterior jaw may endure the greatest change, where the anterior/posterior range is much more sensitive to change.

Furthermore, it was found that in establishing the resting physiologic envelope, during maximal physiologic closing, the lateral pterygoid sites, not investigated in previous studies, were consistently found to be responsive prior to prime mover sites (temporalis anterior or masseter). During maximal physiologic protrusion, the lateral pterygoid consistently demonstrated earlier activation than any other site, and during maximal physiologic opening, it was found to be elevated approximately 30% of the time concurrent with the suprahyoid sites.

This information presents a number of appealing implications as follows.

1. This physiologic resting range of the anterior and posterior mandibular positions may be documented utilizing jaw motion analysis (e.g., Zebris Medical GmbH) when combined with SEMG.
2. The vertical physiologic envelope may be smaller than previously suggested in TMD subjects. The function and activity of the lateral pterygoid suggest that this may be the limiting factor.
3. The anterior border (maximum physiologic protrusion) may be documented if concurrent lateral pterygoid activity is recorded.
4. Caution should be exercised if jaw positioning is considered anterior to a trajectory created by TENS if the lateral pterygoid activity is not measured.
5. Intraoral lateral pterygoid recordings may lend important information in the construction of “pull-forward” appliances for sleep apnea patients to ensure the chosen appliance and/or position is within the physiologic range.
6. Establishing the physiologic borders through the combined use of SEMG and posterior jaw position during a bite registration procedure may allow the clinician to objectively document the range of physiologic rest of the anterior and posterior lower jaw in the vertical and anterior dimensions. Theoretically, this would allow the clinician to ensure the chosen bite registration position is within physiologically tolerable limits, thereby lessening the amount of adaptation that would be required. Within the physiologic zone of rest formed by these 4 positions, the vertical and anterior borders of the envelope of function may be documented for the incisal and posterior mandible in true 3-dimensional fashion to assist the clinician in determining a physiologic interocclusal freeway space and vertical dimension of occlusion.

This and previous works demonstrated tremendous promise for clinical applications for intraoral SEMG of the lateral pterygoid sites if commercially available customized miniature intraoral surface electrodes became available and an established electrode placement protocol based on verified CT sites is tested more thoroughly for clinical applications. Should these parameters be met, use of surface electromyography of the lateral pterygoids would provide noninvasive, functionally diagnostic treatment information not attainable by other means.

In the computed trajectory, one limitation is that functional anatomy dictates that the mandible does not move in a straight line. Therefore, it is seemingly intuitive that any sort of computer generated trajectory is an approximation and the addition of SEMG may be necessary to ensure that any chosen treatment position is within physiologic parameters.

Furthermore, there are obvious biomechanical implications related to the cranial-skeletal system that should be considered. Cranial asymmetries are not uncommon, often associated with craniofacial asymmetries, as well as cerebral asymmetries [49–51]. In view of the fact that the masticatory muscles have attachments to the cranium, if asymmetries or skeletal anomalies exist, muscles are disallowed a biomechanically symmetrical alignment among homologous pairs, predisposing them to dysfunction. Without initially correcting these biomechanical issues, treatment success is likely to be limited with or without the technique or technologies presented.

The author acknowledges some clear limitations. In addition to the aforementioned parameters, the dimensional changes by way of orthodontic or reconstructive or other methods that were considered when referring to the data are speculative only and were not explored and thus, the rigorous research is required to establish validity of the concept. The analyzed individual represents only a convenient sample, and the extrapolation of the results or to different TMD diagnostic groups, should be done with caution. However, the concept does follow basic physiologic principles and appears to be promising.

## Figures and Tables

**Table 1:** Summary of associated mandibular coordinates relative to physiologic parameters, determined by simultaneous monitoring of masticatory muscle activity.

	Right posterior jaw	Incisal point	Right posterior jaw
Physiologic rest	2.5 mm inferior to CO, and 0.3 mm anterior.	4.4 mm inferior to CO, 0.1 anterior and to the right.	2.2 mm inferior to CO, and 0.3 mm anterior.

Maximal physiologic opening	3.6 mm inferior to CO, 0.3 mm to the right, and 1.0 mm inferior to physiologic rest.	6.2 mm inferior to centric occlusion (CO), 0.5 m to the right.	3.3 mm inferior to CO, 0.3 mm to the right of CO, and 1.1 mm inferior to physiologic rest

Maximal physiologic closing	0.1 mm inferior to CO, 0.2 mm to the right of CO, and 2.1 mm superior to physiologic rest.	2.3 mm inferior to CO, 0.2 mm to the right of CO, and 2.1 mm superior to physiologic rest.	0.1 mm inferior to CO, 0.2 mm to the right of CO, and 2.1 mm superior to physiologic rest.

Maximal physiologic protrusion	2.2 mm inferior to CO, 0.2 mm to the right of CO, and 1.7 mm anterior to CO.	4.4 mm inferior to CO, 0.6 mm to the right of CO, 1.2 mm anterior to CO, and 1.0 mm anterior to trajectory.	1.8 mm inferior to CO, 0.2 mm to the right of CO, and 0.9 mm anterior to CO.
